# Pregnancy outcome in women with polycystic ovary syndrome comparing the effects of laparoscopic ovarian drilling and clomiphene citrate stimulation in women pre-treated with metformin: a retrospective study

**DOI:** 10.1186/1477-7827-8-45

**Published:** 2010-05-13

**Authors:** Johannes Ott, Christine Kurz, Kazem Nouri, Stefan Wirth, Elisabeth Vytiska-Binstorfer, Johannes C Huber, Klaus Mayerhofer

**Affiliations:** 1 Department of Gynecologic Endocrinology and Reproductive Medicine, Medical University of Vienna, Vienna, Austria

## Abstract

**Background:**

Ovarian stimulation in women with polycystic ovary syndrome (PCOS) increases the risk for perinatal complications. Ovulation induction by laparoscopic ovarian drilling (LOD) might improve the overall pregnancy outcomes. The aim of our study was to assess the adverse events or effects on pregnancy of LOD and clomiphene citrate (CC) stimulation in patients who received metformin.

**Methods:**

Setting: Academic research institution. We retrospectively analyzed the courses of 40 spontaneous pregnancies after LOD for CC-resistance, 40 pregnancies after CC stimulation, and 40 pregnancies after metformin treatment alone. Patients in the LOD and the CC groups had been pre-treated with Metformin. Primary outcome parameters were: the rate of multiple pregnancies; the rate of early pregnancy losses/miscarriages; the development of gestational diabetes, pregnancy-induced hypertension, and preeclampsia/HELLP-syndrome; premature delivery; and birth weight.

**Results:**

The rate of twin pregnancies did not differ between the CC group (12.5%), the LOD group (7.5%), and the metformin only group (2.5%, p = 0.239). Seventeen women suffered an early miscarriage. There were no differences with regard to the rates of gestational diabetes, pregnancy-induced hypertension, preeclampsia, and preterm delivery. By analyzing all pregnancy complications together, the overall pregnancy complication rate was highest in the CC group (70.0%, 28/40), followed by the LOD group (45.0%, 18/40), and the metformin only group (47.5%, 19/40; p = 0.047).

**Conclusions:**

CC, but not LOD, increases the complication rate in pregnant patients who received metformin.

## Background

Despite the fact that polycystic ovary syndrome (PCOS) remains one of the most common female endocrinopathies, with an incidence of 5-10% in women of reproductive age, the etiology, pathogenesis, and even treatment of infertile women who suffer from PCOS are still the subject of much controversy [1,2]. Clomiphene citrate (CC) is presumed to be the first-line therapy, since it is known to result in higher ovulation, conception, pregnancy, and live-birth rates when compared with metformin; the combination of both drugs did not result in a significant benefit [3,4].

As second- and third-line therapies, gonadotropin stimulation and laparoscopic ovarian drilling (LOD) are recommended for CC-resistant anovulatory PCOS patients. To date, five randomized controlled trials have compared the effectiveness of LOD with that of gonadotropin use, and all demonstrated similar pregnancy and live birth rates for both treatment options [5-8].

Ovarian stimulation has been shown to increase the risk for perinatal complications. Even non-in vitro fertilization assisted reproductive techniques lead to a higher risk for multiple pregnancies, prematurity, low and very low birth weight, transfer to the neonatal intensive care unit and various neonatal morbidity parameters [9]. Thus, it has been proposed that ovulation induction by LOD may improve the overall outcomes of pregnancies in PCOS patients [10]. A comparison of pregnancy outcomes of women with PCOS who had undergone LOD and non-PCOS pregnancy controls demonstrated a significantly higher risk of hypertensive disorders and gestational diabetes mellitus (GDM) for the PCOS group [11]. However, whether LOD might prevent pregnancy complications in women with PCOS remains hypothetical [10].

The aim of our retrospective study was to evaluate the pregnancy outcome after LOD compared to pregnancies after metformin treatment and CC stimulation.

## Methods

### Definition

PCOS was diagnosed according to the revised European Society of Human Reproduction and Embryology (ESHRE) and the American Society for Reproductive Medicine (ASRM) criteria of 2004, which were based on the Rotterdam criteria [2]. CC resistance was defined as the absence of developing follicles after ovarian stimulation with 150 mg clomiphene citrate/day given for five days beginning on the second day of the menstrual cycle.

### Patient collective

In a retrospective cohort study, we included all CC-resistant women with PCOS who underwent LOD at the Department of Gynecology and Obstetrics of the Medical University of Vienna, Austria, between January 2001 and December 2008, and who became spontaneously pregnant within one year after the operation. Of a total of 125 patients who underwent LOD, all necessary data were available for 75 patients (60.0%). Forty women became spontaneously pregnant after LOD and were included in this analysis, whereas women in need of CC stimulation after LOD were excluded (Group 1).

Of a total of 125 PCOS women who had become pregnant after CC stimulation at our department between January 2003 and December 2008, 51 were lost to follow-up (40.8%). Of the remaining 74 patients, 40 age-matched PCOS women were assigned to the CC group (Group 2).

A total of 109 PCOS women had become pregnant after metformin therapy alone at our department between January 2003 and December 2008. In these patients, metformin had been used as a first-line monotherapy for PCOS, since these patients wanted an improvement in both metabolic and reproductive function and were not on a fast track toward becoming pregnant. Thirty-two patients were lost to follow-up (29.4%). Of the remaining 77 patients, 40 age-matched PCOS women were assigned to the metformin group (Group 3). For details on patient characteristics, see Table 1.

**Table 1:** Patient characteristics

		Pregnancies after LOD (n = 40)	Pregnancies after CC stimulation (n = 40)	Pregnancies after metformin therapy only (n = 40)	p
Age (years)*		27.8 ± 4.9	27.5 ± 4.5	27.2 ± 4.6	0.204
Parity	0	38	32	34	0.133
	1	2	7	6	
	2	0	1	0	
Infertility	Prim.^+^	25	23	26	0.781
	Sec.^#^	15	17	14	
BMI (kg/m^2^)		26.9 ± 5.0	28.0 ± 6.0	27.2 ± 5.6	0.656
Luteinizing hormone (IU/l)^+^		13.5 ± 4.9	14.0 ± 4.2	12.2 ± 4.3	0.349
Follicle stimulating hormone (IU/l)^+^		5.9 ± 1.8	6.1 ± 1.7	5.9 ± 1.9	0.917
Testosterone (ng/ml)^+^		0.8 ± 0.4	0.7 ± 0.3	0.8 ± 0.3	0.095
Androstendione (ng/ml)^+^		3.7 ± 1.1	3.3 ± 1.1	3.4 ± 1.1	0.143
Pre-existing arterial hypertension (n, %)^+^		4 (10.0%)	3 (7.5%)	3 (7.5%)	0.897
Abnormal oral glucose tolerance test (n, %)^+^		19 (47.5%)	16 (40.0%)	18 (45.0%)	0.789
Duration of metformin therapy (months)		10.1 ± 2.0	6.9 ± 3.1	3.4 ± 1.0	<0.001^‡^

*Age at conception; ^+^Prim. = primary; ^#^Sec. = secondary; ^‡^statistically significant;

^+ ^before initiation of any PCOS-related treatment

At our department, infertile women with PCOS undergo a first-line treatment with metformin alone in case that there is no urgent need to establish pregnancy, or combined CC stimulation and metformin treatment. Based on individual decisions with regard to the patient's needs, LOD is recommended after a minimum of 3 or a maximum of 6 cycles of CC stimulation has failed to induce ovulation. Thus, all of our patients (Groups 1, 2, and 3) had been treated with metformin for at least two months, with a minimum dose of 1500 mg/day as a standard, first-line therapy before the pregnancy was established. As soon as the pregnancy was diagnosed, the metformin therapy was discontinued.

### Study design

The primary objective of the study was to assess the adverse events or effects on pregnancy of LOD and CC stimulation in patients who received metformin and in patients who received metformin only therapy. The following parameters were analyzed: the rate of multiple pregnancies; the rate of early pregnancy losses/miscarriages; the development of GDM, pregnancy-induced hypertension (PIH), and preeclampsia/HELLP-syndrome; premature delivery; and birth weight.

GDM was diagnosed using the 75 g, 2 h oral glucose tolerance test (OGTT), routinely performed in the third trimester. Patients were diagnosed with GDM if one of the following parameters reached or exceeded the 97.5 percentile: fasting venous blood glucose concentration (4.5 mmol/l), at 1 h (9.1 mmol/l) and 2 h (7.9 mmol/l). The indications for OGTT in the second trimester were as follows: glucose in any of the monthly urine samples; GDM in a previous pregnancy; diabetes in the immediate family; and fetal macrosomy (> 2 standard deviations). PIH was defined as gestational hypertension (blood pressure >140/90 mmHg without proteinuria at a gestational age >20 weeks on two or more occasions at least 6 h apart). Preeclampsia was defined as blood pressure >140/90 mmHg in combination with proteinuria >0.3 g/24 h after 20 weeks gestation. The HELLP-syndrome was defined as hemolysis, elevated liver enzymes (serum LDH ≥ 600 IU/L or total bilirubin ≥ 1.2 mg/dL), and low platelet count (≤ 100.000 cells/μL). Premature delivery was considered as delivery between the 22nd and 37th week of gestation.

Furthermore, we evaluated the following parameters from retrospective chart review: age; body mass index (BMI); sexual hormone levels including luteinizing hormone (LH), follicle stimulating hormone (FSH), testosterone, androstenedione, and oral glucose tolerance test as measured at the initial visit before any PCOS-related medication; diabetes mellitus; and pre-existing essential arterial hypertension and liver diseases before pregnancy was established.

### Surgical technique of laparoscopic ovarian drilling

LOD was achieved with standard laparoscopy under general anesthesia. The procedure was performed after establishing a CO_2 _pneumoperitoneum. The laparoscope was inserted through the umbilicus using a 5 mm trocar. Under direct supervision, two 5-mm adjuvant trocars were inserted subsequently.

Thirty-one of the reported patients who underwent LOD were treated with bilateral LOD using a monopolar electrocoagulation technique, as previously reported [12]. Nine patients were operated with a monopolar hook electrode (GK 375R, B. Braun Aesculap, Maria Enzersdorf, Austria) and 5-10 incisions of 2-3 mm length in the ovarian capsule.

The study was performed in accordance with the "Good Scientific Practice Standards" set forth by the Medical University of Vienna, which are based on the ethical standards of the revised Helsinki Declaration of 2008. The study was approved by the Institutional Review Board of the Medical University of Vienna (IRB number: 1121/2009).

### Statistical analysis

Variables are described by frequencies and mean ± standard deviation (SD). Statistical analysis was performed using the chi-square test, the likelihood-quotient, univariate variance analysis, and the Kruskal-Wallis test. Differences were considered statistically significant if p < 0.05. Statistical analysis was performed using SPSS 15.0.1 for Windows (SPSS Inc., 1989-2006).

## Results

### Patient characteristics

Details about patient characteristics are listed in Table 1. The duration of metformin therapy before pregnancy was longest in the LOD group, followed by the CC group (p < 0.001). For the LOD patients, the time between the operation and the last menstrual bleeding before conception was 6.1 ± 2.9 months. These patients had been stimulated for a minimum of 3 and a maximum of 6 cycles before the operation. In patients who became pregnant after CC stimulation, the mean number of stimulations was 1.6 ± 0.8. Pregnancies were established after a stimulation with 50 mg and 100 mg in 30 and 10 patients, respectively. None of the hormonal parameters were found to differ between the three groups. No woman had suffered from diabetes mellitus or any kind of liver disease pre-existing before pregnancy was established. The incidences of essential arterial hypertension were distributed equally (p > 0.05).

### Pregnancy outcome

Details on early pregnancy courses and outcomes can be seen in Table 2. There was no case of ovarian hyperstimulation syndrome. The rate of twin pregnancies was highest in the CC group (12.5%), followed by the LOD and the metformin only groups (7.5% and 2.5%, respectively). However, the differences were not statistically significant (p = 0.239). All other patients had singleton pregnancies. Seventeen women suffered from an early miscarriage. In two patients, the pregnancy was terminated because of fetal malformations: one 26-year-old woman from the LOD group for fetal anencephaly in her 17^th ^gestational week; and one 26-year-old woman from the CC group for fetal Down syndrome in her 16^th ^gestational week. One woman from the LOD group suffered a late pregnancy loss in her 22^nd ^gestational week.

**Table 2:** Early pregnancy outcome

	Pregnancies after LOD (n = 40)	Pregnancies after CC stimulation (n = 40)	Pregnancies after metformin therapy only (n = 40)	p
Twin pregnancies	3/40 (7.5%)	5/40 (12.5%)	1/40 (2.5%)	0.239
Early miscarriage (<20^th ^week)	4/40 (10.0%)	8/40 (20.0%)	5/40 (12.5%)	0.410
Pregnancy termination for fetal malformations	1/36 (2.8%)	1/32 (3.1%)	0	0.441
Late pregnancy loss (20^th ^- 24^th ^week)	1/35 (2.9%)	0	0	0.331

Table 3 shows details outcomes of the 100 pregnancies remaining after the 24^th ^gestational week. The highest rate of preterm deliveries before the completed 37^th ^week of gestation was found for the CC group (19.4%), followed by the LOD and metformin only groups (8.8% and 5.7%, respectively). Again, the differences were not significant (p = 0.200). One male child was delivered by caesarean section in the 25^th ^week of gestation due to preterm, prelabor rupture of membranes and intra-amniotic infection. The child died at the neonatal intensive care unit a few days after delivery. No other children suffered from any morbidities in the postpartum period.

**Table 3:** Late pregnancy outcome

	Pregnancies after LOD (n = 34)	Pregnancies after CC stimulation (n = 31)	Pregnancies after metformin therapy only (n = 35)	p
PIH*	1 (2.9%)	4 (12.5%)	4 (11.4%)	0.262
Preeclampsia/HELLP syndrome	4 (11.8%)	4 (12.5%)	1 (2.9%)	0.238
GDM^+^	10 (29.4%)	10 (31.3%)	11 (31.4%)	0.980
IGDM^#^	5 (14.7%)	5 (15.6%)	5 (14.3%)	0.988
Preterm delivery <37^th ^gestational week	3 (8.8%)	6 (19.4%)	2 (5.7%)	0.200

*PIH = pregnancy induced hypertension; ^+^GDM = gestational diabetes mellitus; ^#^IGDM = insulin-dependent gestational diabetes mellitus; ^‡^statistically significant

The mode of delivery was a caesarean section in 38.2% (13/34), 51.6% (16/31), and 34.3% (12/35) for groups 1, 2 and 3, respectively. Birth weight did not differ between groups 1, 2, and 3 (3243.1 ± 628.9 vs. 3261.3 ± 740.9 vs. 3073.3 ± 631.9, respectively; p = 0.253).

### Overall pregnancy complication rate

By analyzing all pregnancy complications together, i.e., early miscarriage, fetal malformations, late pregnancy loss, development of PIH, preecmlampsie/HELLP-syndrome, and GDM, as well as preterm delivery, the following distribution was found: 70.0% (28/40) for the CC group; 45.0% (18/40) for the LOD group; and 47.5% (19/40) for the metformin only group (p = 0.047).

## Discussion

In this retrospective study of 120 pregnancies after different treatment regimes for infertility in PCOS women, we found no significant differences in pregnancy outcomes, except for the overall complication rate. However, there was some trend toward higher early miscarriage, twin pregnancy, and preterm delivery rates for pregnancies after CC stimulation.

With regard to general patient characteristics, such as age, parity, type of infertility, body mass index, hormonal parameters and the incidence of pre-existing arterial hypertension, there were no differences between the three study groups. However, we observed a significantly longer intake of metformin in the LOD group, followed by the CC and metformin only groups. This is due to the fact that metformin is given as the standard first-line therapy for many anovulatory, young PCOS patients. In the event that metformin therapy failed to induce ovulation, women were subsequently treated with CC stimulation for a maximum of six cycles. Only CC-resistant women underwent LOD. However, metformin therapy was continued during CC stimulation and before and after LOD. Thus, LOD patients had the longest metformin intake.

To our knowledge, this is the first study to compare pregnancy outcomes after different treatment regimes, including LOD for anovulatory PCOS women. To date, only one trial has focused on pregnancy outcomes after LOD compared to pregnancies after spontaneous ovulation and conception in healthy women. The authors of that study reported a significantly higher risk of hypertensive disorders and GDM in the LOD group [11]. In our patient collective, similar incidences of GDM of about 30% were found for the three study groups. Nonetheless, the overall GDM rate of 30.7% reflects the increased risk for GDM in patients with PCOS compared to healthy women [10]. We observed a trend toward a higher risk of PIH and preeclampsia/HELLP syndrome that was somewhat more profound in the CC group in our study collective. However, these data failed to reach statistical significance (p > 0.2). Whether this is caused by possibly too small sample sizes cannot be concluded from this data. It is known that, even after LOD, the rates of pregnancy-induced hypertensive disorders are increased when compared to healthy controls [11].

Although statistically not significant, we observed the highest rate of twin pregnancies after CC stimulation. This is in accordance with the recent literature reporting that CC stimulation leads to slightly increased rates of multiple pregnancies when compared to pregnancies after spontaneous conception. The multiple pregnancy rate after CC stimulation in PCOS women has been reported to be less than 10% [2,10]. The rate of twin pregnancies after CC stimulation of 12.5% in our study may be considered similar.

Rates of early pregnancy loss of up to 40% have been reported for PCOS women [13]. As mentioned, treatment with ovulation-inducing medications might be responsible for this increased risk for early pregnancy loss. This is in accordance with the observation that CC stimulation leads to a significantly higher incidence of miscarriage compared to spontaneous pregnancies in subfertile women [14]. Similarly, we found the highest rate of early miscarriage in the CC group in our study (20%), whereas rates of about 10% were observed in the LOD and metformin only groups.

The rate of preterm deliveries before the 37^th ^completed week of gestation was highest for pregnancies after CC stimulation (about 20%). Again, these differences were not significant, perhaps due to the small sample size. There were no differences in birth weight and neonatal morbidity or mortality.

We analyzed the overall risk for any kind of pregnancy complication, and found a significantly higher risk in the CC group (70%) than in the other groups (about 45%). This might be an indication that pregnancy after LOD is somewhat superior to that after CC stimulation in PCOS patients. However, these data have no validity with regard to patients who have undergone LOD, but who are still in need of CC stimulation subsequently in order to become pregnant. In further studies, we will focuse on this group of patients.

The additive effect of combined metformin and CC or metformin and LOD may be a confounding factor. We consider this a study weakness.

## Conclusions

CC, but not LOD for CC resistance, somehow increases the complication rate in pregnant patients who received metformin. However, since our study does not demonstrate a significant benefit of LOD treatment for CC resistance compared to CC stimulation concerning the major outcome parameters except for the overall complication rate, LOD cannot be recommended as a first-line therapy for anovulatory PCOS women under metformin treatment. One has to consider that LOD might lead to a decrease in the ovarian reserve due to a surgical destruction of the ovarian capsule and/or to adhesion formation, both possibly resulting in impairment of fertility. Thus, LOD must remain a second- or third-line treatment option [15-18]. Currently, not only ovulation and pregnancy rates but also pregnancy outcome, live birth rate, and baby take-home rate are considered the ultimate clinical endpoints for studies on infertility treatment. Thus, larger, prospective, randomized trials are necessary in order to compare pregnancy outcomes after different treatment regimes for anovulatory infertile PCOS women and to finally evaluate the value of LOD.

## Competing interests

The authors declare that they have no competing interests.

## Authors' contributions

All authors conceived and designed the study. JO, SW, CK, JCH, and KM supervised the data collection, assisted in the analysis, and drafted the manuscript; JO and SW assisted in collection and maintenance of the data; all authors assisted in conception, design, and analysis, and edited the manuscript. All authors read and approved the final manuscript.
